# Supplementary material for: Wearable data reveals distinct characteristics of individuals with persistent symptoms after a SARS-CoV-2 infection
Source: NPJ Digit Med. 2025 Mar 19;8:167. doi: 10.1038/s41746-025-01456-x (PMC11920215; doi:10.1038/s41746-025-01456-x)
Supplement: Supplementary file 1 — Supplementary Information [file 41746_2025_1456_MOESM1_ESM.pdf]

SUPPLEMENTARY INFORMATION: WEARABLE DATA REVEALS DISTINCT CHARACTERISTICS OF INDIVIDUALS WITH PERSISTENT SYMPTOMS AFTER A SARS-COV-2 INFECTION

Supplementary Table I. Information on other diseases and conditions of all studied cohorts. See Materials and Methods (Section IV) for further details on the specifics of the assignments.

	COVID-19 [+][1]	COVID-19 [-]	COVID-19 [+]NS[2]	COVID-19 [+]PS[3]	M-COVID-19 [+]NS[4]	M-COVID-19 [-]	M-COVID-19 [+]PS[5]
N	7,828	12,987	7,691	137	150	150	50
% answered	89.9***	87.7	89.9***	88.3	90.0	89.3	90.0
No disease in the past 12 months [%]	48.5***	44.3	49.0***	24.8***	54.1	41.0	31.1*
Allergies [%]	30.4	30.0	30.0	50.4***	25.2	32.1	46.7**
Hypertension [%]	18.2***	22.6	18.1***	23.1	16.3	25.4	20.0
Asthma [%]	9.3	9.3	9.0	24.0***	11.1	11.9	22.2
Increased blood lipid/ cholesterol levels [%]	10.4***	12.8	10.3***	15.7	8.1	12.7	17.8
Chronic Bronchitis [%]	2.1***	3.1	2.0***	6.6**	1.5	1.5	8.9*
Other [%]	10.5***	13.9	10.3***	19.8*	10.4	9.7	22.2
Mental Illness Diagnose [%]	19.5**	22.2	19.2††	39.7***	15.6	20.1	44.4***

[1] $COVID-19[+]$ vs. $COVID-19[-]$; [2] $COVID-19[+]NS$ vs. $COVID-19[-]$; [3] $COVID-19[+]PS$ vs. $COVID-19[+]NS$; [4] $M-COVID-19[+]NS$ vs. $M-COVID-19[-]$;[5] $M-COVID-19[+]PS$ vs. $M-COVID-19[+]NS$. p-values: *p ¡ 0.05, **p ¡ 0.01, ***p ¡ 0.001.

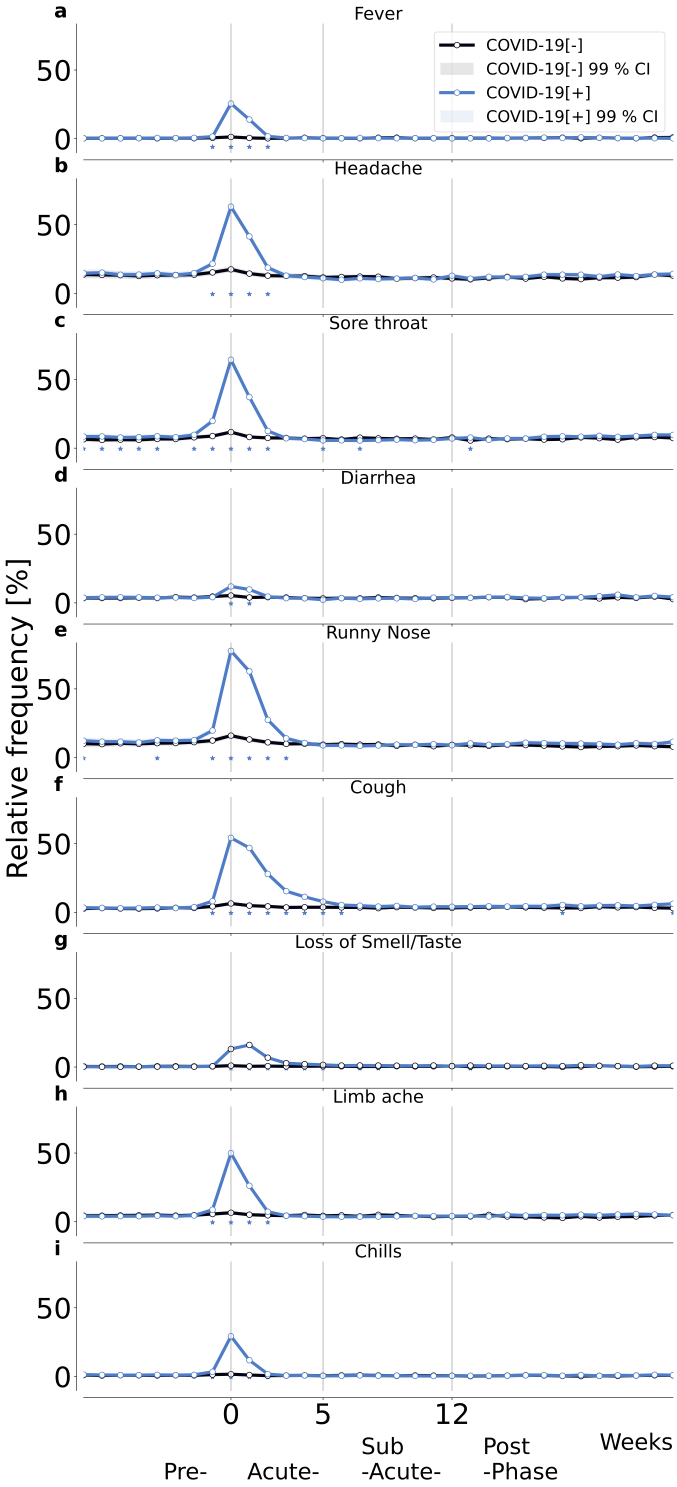

Supplementary Figure 1. Relative frequency of symptoms relative to week of reported SARS-CoV-2 test for positive cohort in blue ($COVID-19[+]$) and negative control cohort ($COVID-19[-]$) in black. The symptoms are fever (a), headache (b), sore throat (c), diarrhea (d), runny nose (e), cough (f), loss of smell/taste (g), limb ache (h), and chills (i). Shading indicates the 99% confidence interval, i.e, 2.576 times the standard error of a binomial distribution. Asterisks indicate significant differences between the cohorts using a two-sided two proportion z-test with a significance level of 0.01.

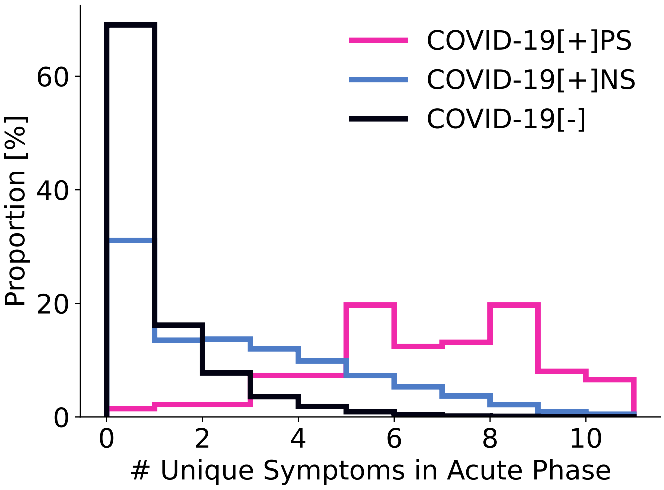

Supplementary Figure 2. Distribution of number of unique reported symptoms during acute phase of the infection (0-4 weeks to the reported SARS-CoV-2 test). We show the proportion of individuals per number of unique symptoms during the acute-phase of the infection for the persistent symptoms in pink ($COVID-19[+]PS$), positive control in blue ($COVID-19[+]NS$) and negative control cohort in black ($COVID-19[-]$).

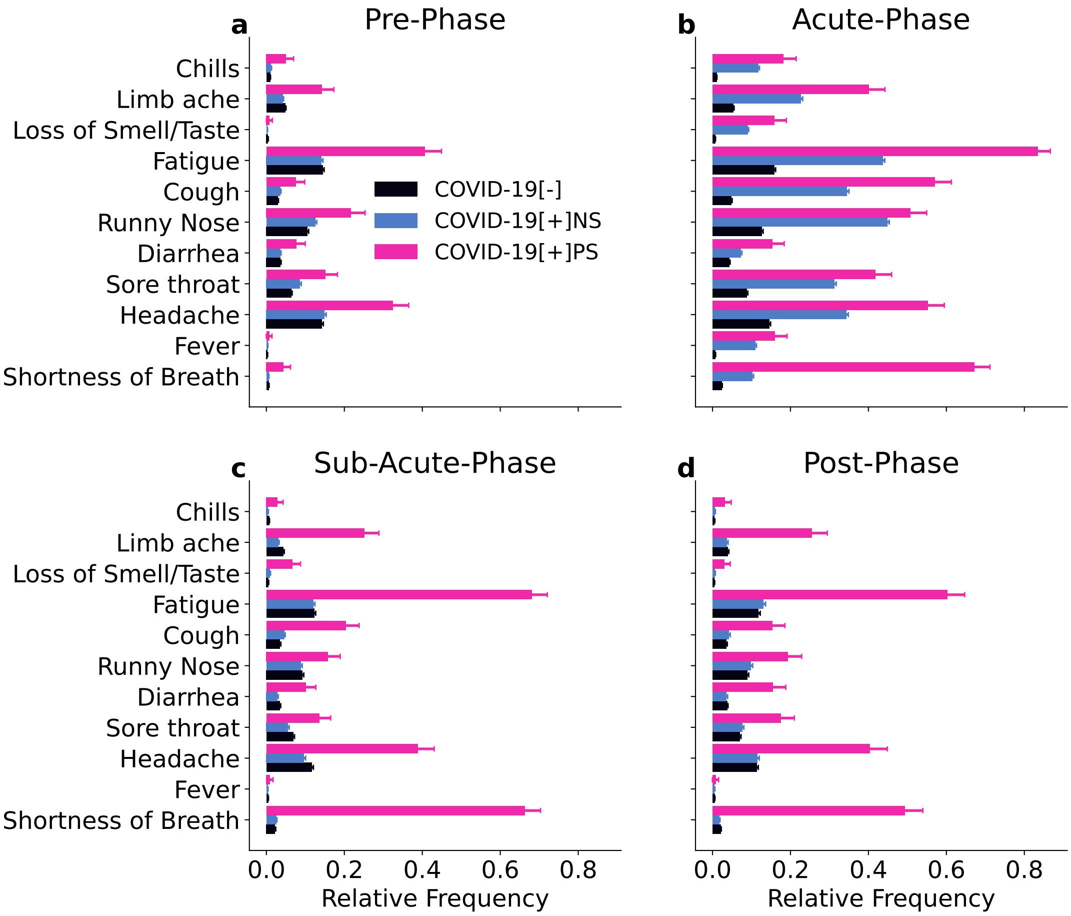

Supplementary Figure 3. Relative frequency per symptom. For pre- (a), acute- (b), sub-acute (c) and post (d) phase we show the relative frequency per reported symptom for all three groups in the matched cohort: negative match in black ($M - COVID - 19[-]$), positive match in blue ($M - COVID - 19[+]NS$) and persistent symptoms match in pink ($M - COVID - 19[+]PS$).

Data Feature	Num Individuals	Avg Epoch Length (seconds)	Num Data Points
HeartRate	283,940	44.78	36,995,160,704
HeartRateResting	232,954	62.21	15,694,872,710
Steps	322,244	201.78	6,226,707,247
MetabolicEquivalent	65,120	626.61	3,170,317,000
RespirationRateSleep	23,851	60.96	2,634,561,312
ActivityType	185,674	997.62	1,961,217,140
ActivitySedentaryBinary	133,566	841.70	1,908,156,825
CoveredDistance	133,212	214.21	1,341,050,259
ActiveBurnedCalories	156,420	315.17	1,185,902,273
ActivityTypeDetail1	176,132	283.36	1,000,501,030
ActivityTypeDetail2	175,710	278.91	998,783,694
WalkBinary	173,608	244.98	944,669,898
SleepInBedBinary	200,378	1817.85	910,357,167
SPO2	22,073	148.18	604,492,043
HeartRateRestingHourly	231,666	3600.00	600,775,921
SleepStateBinary	200,390	2487.74	569,761,356
SleepBinary	204,838	2526.73	568,004,314
SleepLightBinary	129,553	1445.87	496,655,002
ActivityLowBinary	135,437	881.75	423,878,964
RespirationRate	14,216	60.94	290,859,715
SleepDeepBinary	129,355	1365.70	195,606,255
SleepAwakeBinary	151,975	356.34	176,250,333
ActivityMidBinary	135,237	381.29	167,815,161
SleepREMBinary	66,807	1042.99	82,603,650
Speed	10,802	60.00	74,859,514
ElevationGain	125,398	875.05	69,601,558
ActivityHighBinary	134,714	536.57	60,984,492
FloorsClimbed	125,292	1200.23	49,408,478
RunBinary	133,604	514.14	48,051,885
ElevationLoss	9,743	60.00	45,900,565
BurnedCalories	27,735	387.45	43,779,030
Temperature	3,439	60.00	38,795,338
RestBinary	22,634	2622.64	35,088,021
Cadence	8,517	60.00	31,511,617
CoveredDistanceRun	72,983	293.67	31,163,328
DoffedBinary	12,591	3194.94	27,098,222
InterbeatIntervals	3,567	60.00	17,471,943
CoveredDistanceBike	59,750	621.39	16,973,284
CoveredDistanceWalk	5,862	60.00	11,726,427
Rmssd	366	300.00	10,322,810
PowerInWatts	2,551	60.00	10,023,305
SleepInterruptionBinary	40,356	211.77	9,769,554
BikeBinary	131,977	2975.33	8,051,393
TransportBinary	24,649	1631.65	7,205,968
ActiveBinary	111,246	2690.53	5,039,954
ActivityIntensity	11,504	2069.29	2,767,883
CoveredDistanceActive	3,701	60.00	2,390,199
CoveredDistanceBikeManual	20,120	3459.49	318,848
CoveredDistanceRunManual	22,539	3063.90	193,564
SleepAwakeAfterWakeUpBinary	366	707.67	116,105
SleepLatencyBinary	365	604.18	107,391
Weight	1,686	1829.74	21,035
Height	266	60.00	2,070

Supplementary Table II. Summary statistics of wearable data features, including the number of individuals, average epoch length in seconds, and the number of data points for each feature.

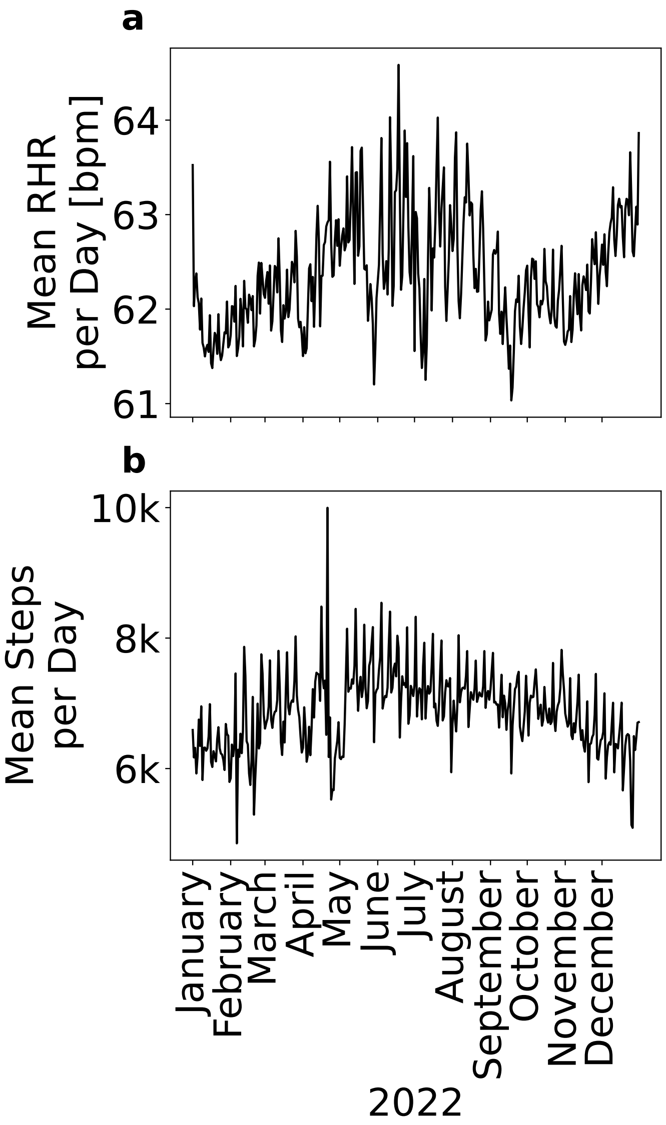

Supplementary Figure 4. Seasonal trends in wearable data. Exemplary timeseries of mean RHR per day (a) and mean steps per day (b) over the CDA Population for one example year (2022) and one example donation-source (Apple).

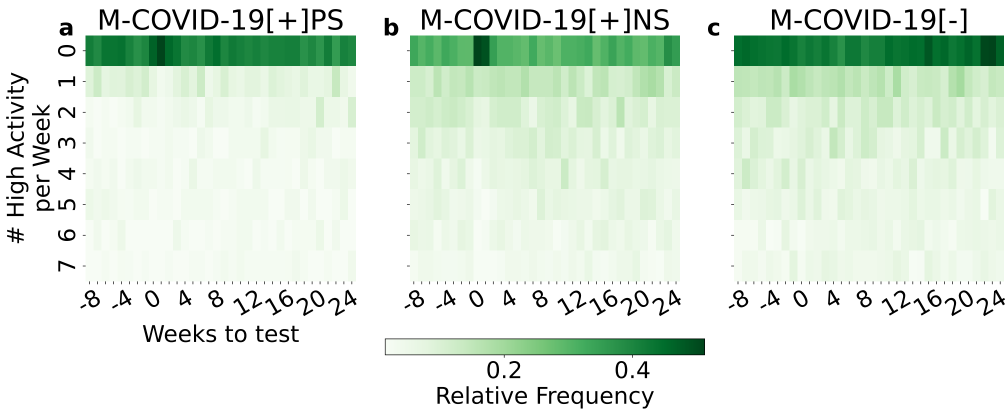

Supplementary Figure 5. High activity profiles of the *matched cohort*. Relative frequency of the number of high activity days per week relative to the SARS-CoV-2 test date for $M-COVID-19[+]PS$ (a), $M-COVID-19[+]NS$ (b) and $M-COVID-19[-]$ (c). A day is defined as a high activity day when the corresponding number of steps per day exceed one standard deviation over the seasonal mean taken over the whole set of users in the Corona Data Donation Project (CDA Population).

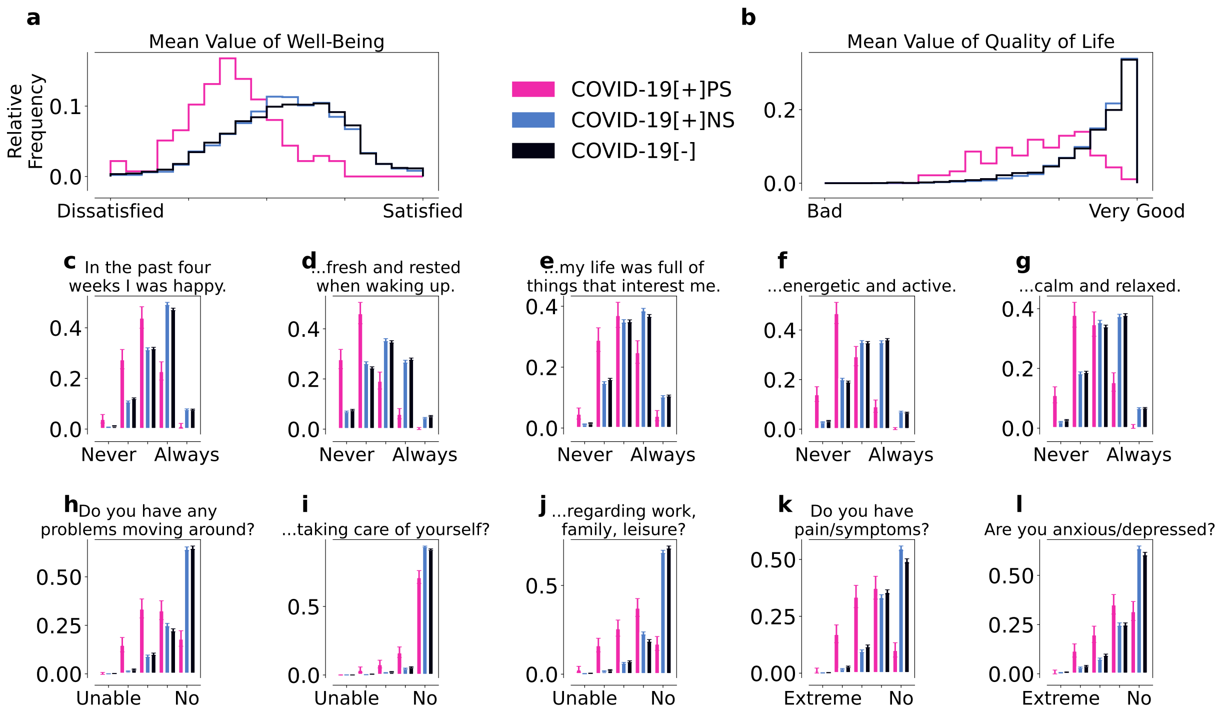

Supplementary Figure 6. Answers to WHO-5 wellbeing and EQ-5D questionnaires before the reported SARS-CoV-2 test for the persistent symptoms (pink), positive control (blue) and negative control cohort (black). WHO-5 wellbeing (a,c-g) and modified EQ-5D/QoL (b,h-l) for the $COVID-19[+]PS$ (pink), $COVID-19[+]NS$ (blue), and $COVID-19[-]$ (black) cohorts before the reported SARS-CoV-2 test. The individual WHO-5 and modified EQ-5D scores were both averaged to obtain the overall wellbeing (a) and QoL (b) scores, respectively. Overall, $COVID-19[+]PS$ individuals reported more issues with wellbeing (c-g) and QoL (h-l) than the control cohorts. Error bars indicate standard errors.

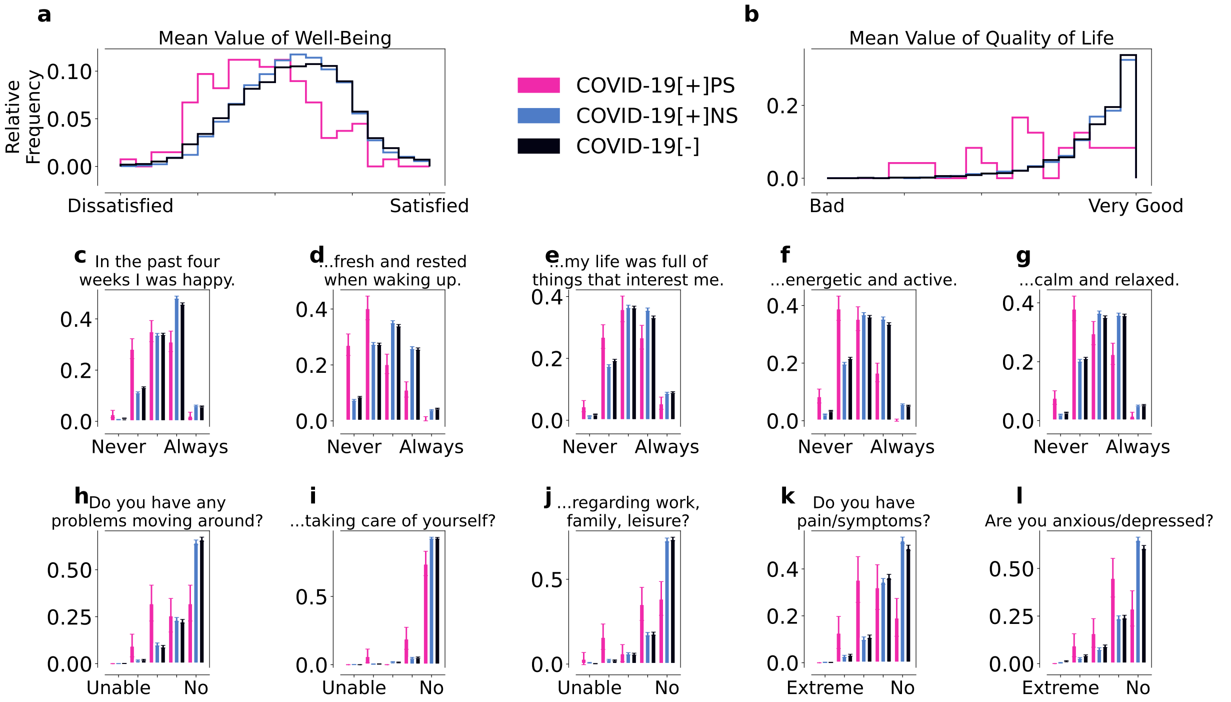

Supplementary Figure 7. Answers to WHO-5 wellbeing and EQ-5D questionnaires after the reported SARS-CoV-2 test for the persistent symptoms (pink), positive control (blue) and negative control cohort (black). WHO-5 wellbeing (a,c-g) and modified EQ-5D/QoL (b,h-l) for the $COVID-19[+]PS$ (pink), $COVID-19[+]NS$ (blue), and $COVID-19[-]$ (black) cohorts after the reported SARS-CoV-2 test. The individual WHO-5 and modified EQ-5D scores were both averaged to obtain the overall wellbeing (a) and QoL (b) scores, respectively. Overall, $COVID-19[+]PS$ individuals reported more issues with wellbeing (c-g) and QoL (h-l) than the control cohorts. Error bars indicate standard errors.

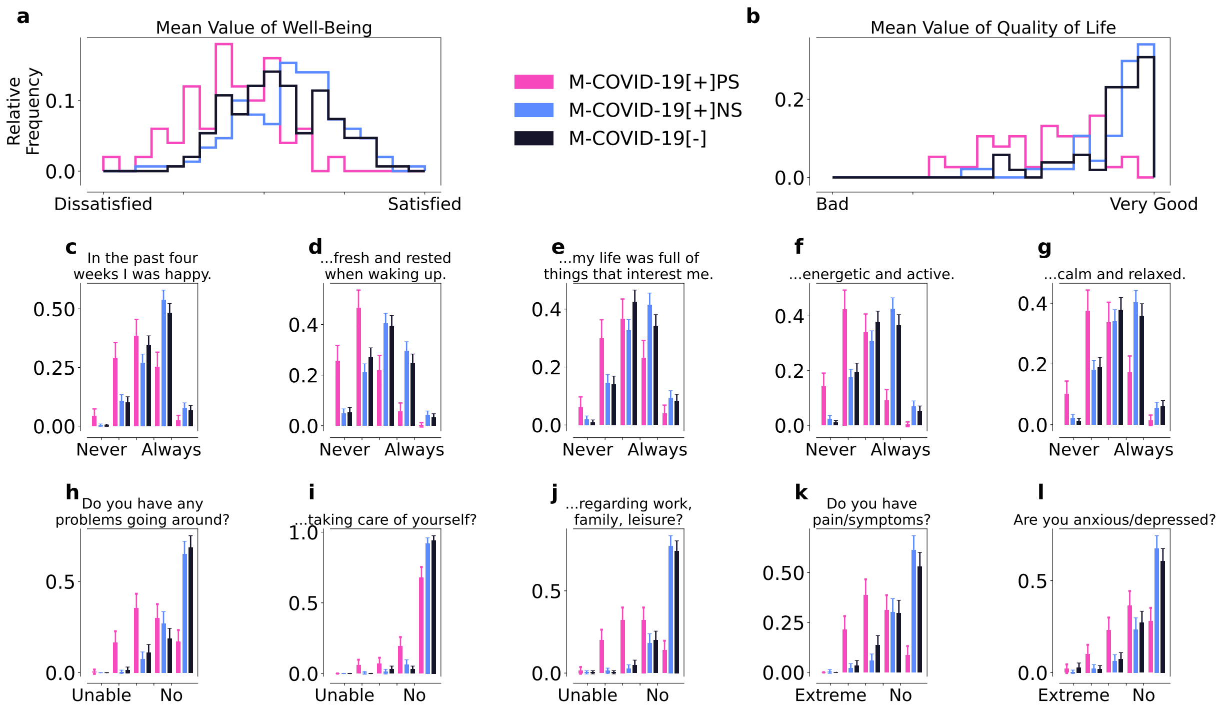

Supplementary Figure 8. Answers to WHO-5 wellbeing and EQ-5D questionnaires for the $M-COVID-19[+]PS$ (pink), $M-COVID-19[+]NS$ (blue) and $M-COVID-19[-]$ cohort (black). WHO-5 wellbeing (a,c-g) and modified EQ-5D/QoL (b,h-l) for the $COVID-19[+]PS$ (pink), $COVID-19[+]NS$ (blue), and $COVID-19[-]$ (black) cohorts after the reported SARS-CoV-2 test. The individual WHO-5 and modified EQ-5D scores were both averaged to obtain the overall wellbeing (a) and QoL (b) scores, respectively. Overall, $COVID-19[+]PS$ individuals reported more issues with wellbeing (c-g) and QoL (h-l) than the control cohorts. Error bars indicate standard errors.

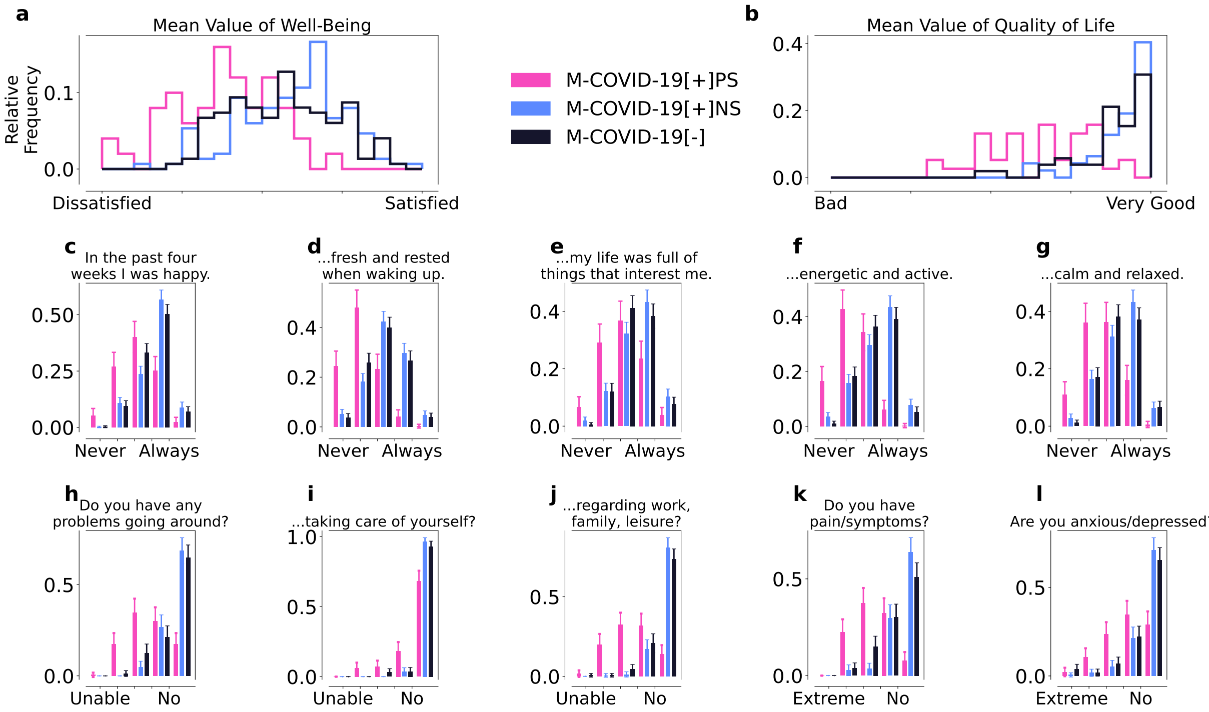

Supplementary Figure 9. Answers to WHO-5 wellbeing and EQ-5D questionnaires before the reported SARS-CoV-2 test for the $M - COVID - 19[+]PS$ (pink), $M - COVID - 19[+]NS$ (blue) and $M - COVID - 19[-]$ cohort (black). WHO-5 wellbeing (a,c-g) and modified EQ-5D/QoL (b,h-l) for the $COVID - 19[+]PS$ (pink), $COVID - 19[+]NS$ (blue), and $COVID - 19[-]$ (black) cohorts after the reported SARS-CoV-2 test. The individual WHO-5 and modified EQ-5D scores were both averaged to obtain the overall wellbeing (a) and QoL (b) scores, respectively. Overall, $COVID - 19[+]PS$ individuals reported more issues with wellbeing (c-g) and QoL (h-l) than the control cohorts. Error bars indicate standard errors.

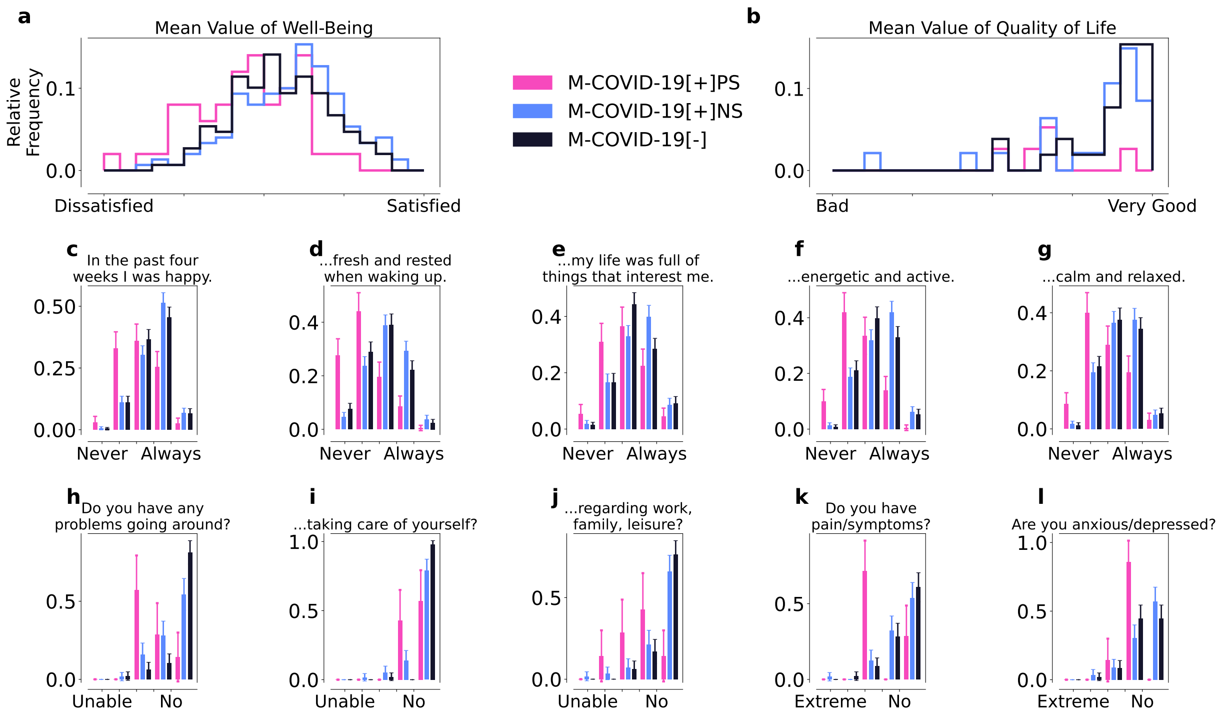

Supplementary Figure 10. Answers to WHO-5 wellbeing and EQ-5D questionnaires after the reported SARS-CoV-2 test for the $M - COVID - 19[+]PS$ (pink), $M - COVID - 19[+]NS$ (blue) and $M - COVID - 19[-]$ cohort (black). WHO-5 wellbeing (a,c-g) and modified EQ-5D/QoL (b,h-l) for the $COVID - 19[+]PS$ (pink), $COVID - 19[+]NS$ (blue), and $COVID - 19[-]$ (black) cohorts after the reported SARS-CoV-2 test. The individual WHO-5 and modified EQ-5D scores were both averaged to obtain the overall wellbeing (a) and QoL (b) scores, respectively. Overall, $COVID - 19[+]PS$ individuals reported more issues with wellbeing (c-g) and QoL (h-l) than the control cohorts. Error bars indicate standard errors.

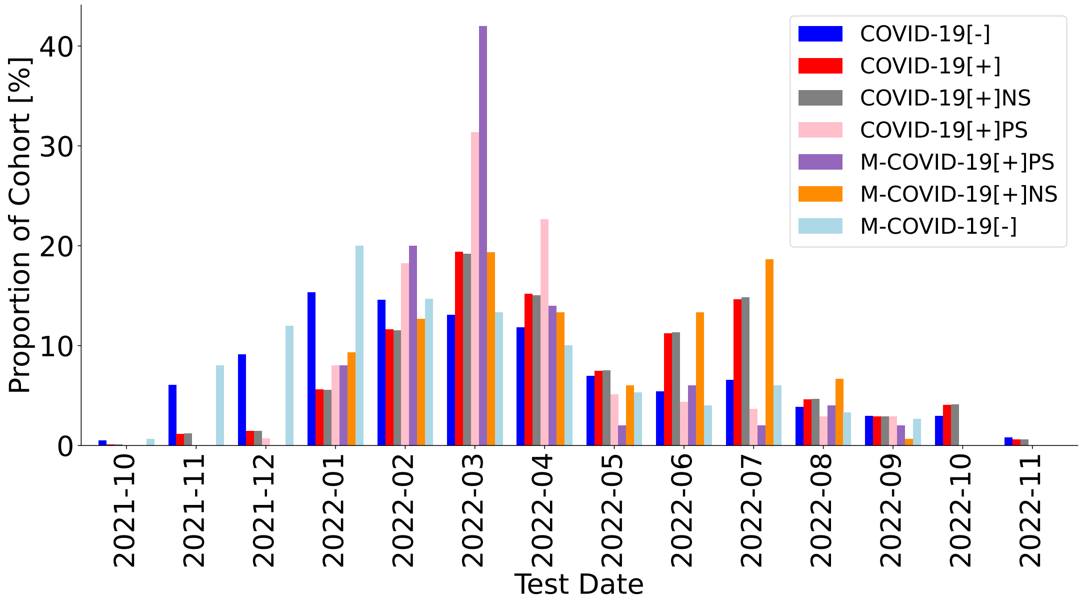

Supplementary Figure 11. Distribution of reported SARS-CoV-2 testdates of studied cohorts. We show per month ranging from October 2021 to November 2022 the proportion of individuals who indicated their SARS-CoV-2 test in the respective month for the COVID-19[-] (negative, black), COVID-19[+] (positive, red), COVID-19[+]NS (positive control, grey), COVID-19[+]PS (persistent symptoms, pink), M-COVID-19[+]PS (persistent symptoms match, blue), M-COVID-19[+]NS (positive match, orange), and M-COVID-19[-] (negative match cohort, light black).

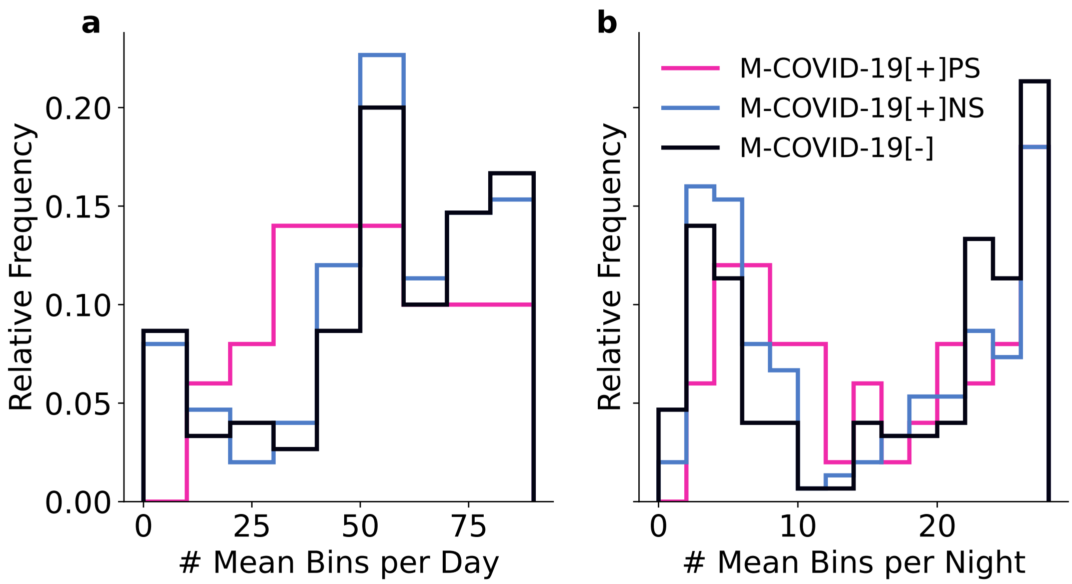

Supplementary Figure 12. Distribution of time-worn of wearable devices. Number of 15 minute heart rate measurements as a proxy for wearing time for the three age and sex matched cohorts M-COVID-19[+]PS (persistent symptoms match, blue), M-COVID-19[+]NS (positive match, pink), and M-COVID-19[-] (negative match cohort, black) in 24 hours (a) and during night time from 11pm until 6am (b).

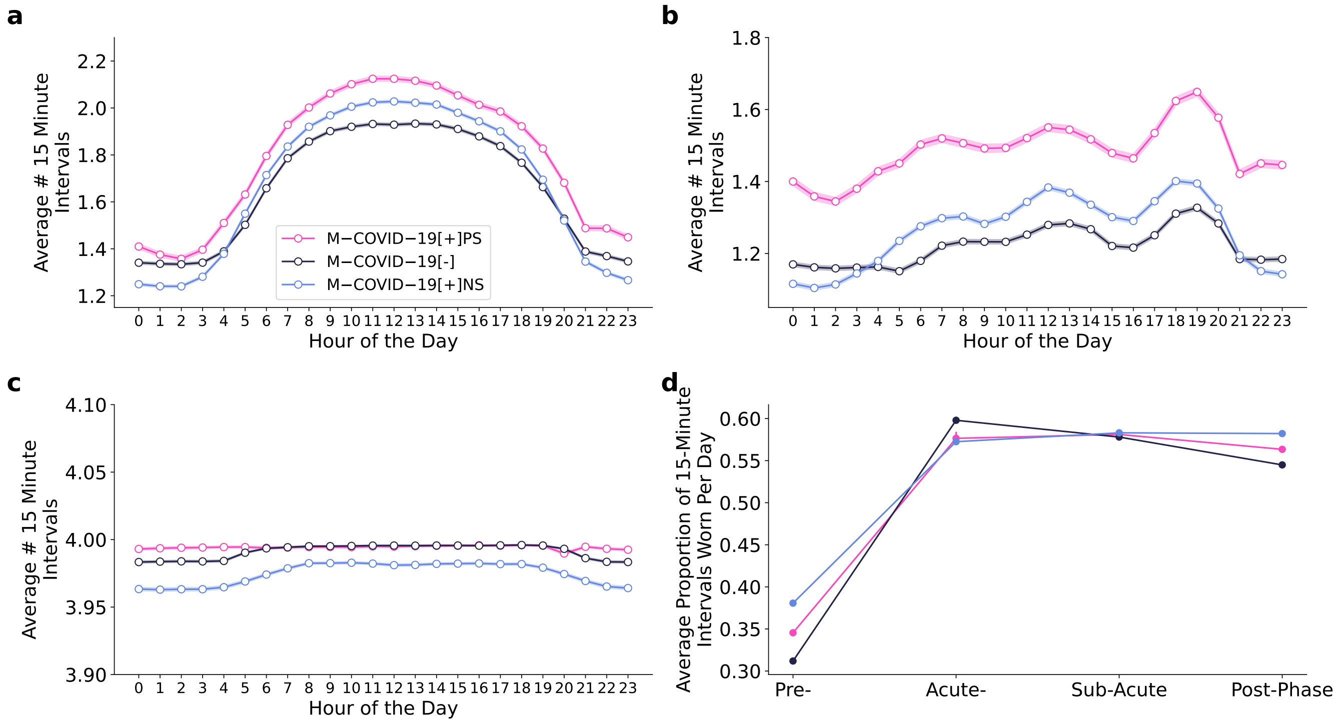

Supplementary Figure 13. Distribution of time-worn of wearable devices per hour of day. Number of 15 minute heart rate (a), resting heart rate (b), and steps (c) measurements as a proxy for wearing time for the three age and sex matched cohorts M-COVID-19[+]PS (persistent symptoms match, blue), M-COVID-19[+]NS (positive match, pink), and M-COVID-19[-] (negative match cohort, black). (d) Average proportion of 15 minute heart rate intervals as a proxy for average time worn per day per phase relative to the reported SARS-CoV-2 test for the three age and sex matched cohorts M-COVID-19[+]PS (persistent symptoms match, blue), M-COVID-19[+]NS (positive match, pink), and M-COVID-19[-] (negative match cohort, black).

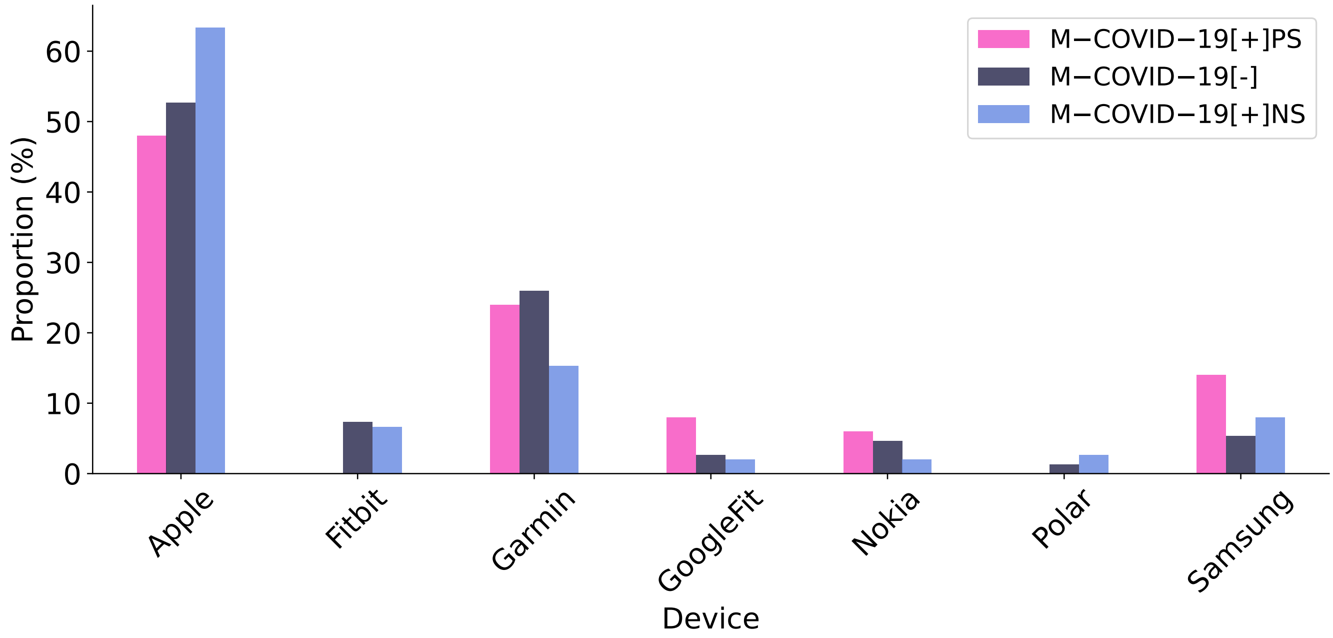

Supplementary Figure 14. Proportion of individuals per wearable device for the three age and sex matched cohorts M-COVID-19[+]PS (persistent symptoms match, blue), M-COVID-19[+]NS (positive match, pink), and M-COVID-19[-] (negative match cohort, black).
